# Evaluating chronic bone and soft tissue infections with [^68^Ga]Ga-Pentixafor PET/CT: a head-to-head comparison with scintigraphy

**DOI:** 10.1007/s00259-025-07749-3

**Published:** 2026-01-27

**Authors:** Dilara Denizmen Zorba, Duygu Has Simsek, Yasemin Sanli, Muhammet Ibrahim Karacam, Omer Naci Ergin, Arif Atahan Cagatay, Fikret Buyukkaya, Serkan Kuyumcu

**Affiliations:** 1 Istanbul Faculty of Medicine, Department of Nuclear Medicine, Istanbul University, Istanbul, Turkey; 2 Istanbul Faculty of Medicine, Department of Orthopedics and Traumatology, Istanbul University, Istanbul, Turkey; 3 Istanbul Faculty of Medicine, Department of Infectious Diseases and Clinical Microbiology, Istanbul University, Istanbul, Turkey

**Keywords:** [^68^Ga]Ga-Pentixafor PET/CT, CXCR4, Labelled leucocyte scintigraphy, Chronic infection, Periprosthetic infection, Diabetic foot infection

## Abstract

**Purpose:**

Chronic bone and soft tissue infections pose a diagnostic challenge, as conventional imaging techniques often show limited sensitivity and specificity. Given that CXCR4 chemokine receptors are expressed on lymphocytes, key mediators of chronic inflammatory response, we hypothesized that CXCR4-targeted imaging may offer enhanced diagnostic accuracy. Accordingly, this study aimed to evaluate the diagnostic performance of [^68^Ga]Ga-Pentixafor PET/CT in comparison with conventional 3-phase bone scintigraphy and [^99m^Tc]Tc-HMPAO-labelled leucocyte scintigraphy in patients with suspected chronic bone and soft tissue infections.

**Methods:**

In this retrospective single-centre study, we included patients who underwent both conventional scintigraphic imaging and [^68^Ga]Ga-Pentixafor PET/CT. Asymptomatic prostheses, orthopaedic implants, and diabetic foot regions within the same cohort served as controls. Two nuclear medicine specialists, blinded to patient data, evaluated imaging findings in consensus visually and quantitatively. Final diagnoses were confirmed by microbiological culture and/or histopathology for infected sites, and by clinical and radiological follow-up for non-infected control sites.

**Results:**

A total of 20 patients with 25 suspected infectious foci and 14 control sites were evaluated. Of the 25 sites, 21 were confirmed to be infected; no infections were observed in the control sites. Scintigraphy correctly identified 19 infection sites (sensitivity: 90%, specificity: 83%), while [^68^Ga]Ga-Pentixafor PET/CT was positive in all 21 infection sites, but demonstrated two false-positives (sensitivity: 100%, specificity: 89%). PET/CT showed higher overall accuracy (95% vs. 87%), although this difference did not reach statistical significance (*p* = 0.07).

**Conclusion:**

[^68^Ga]Ga-Pentixafor PET/CT was accurate in detecting BSTIs, suggesting potential utility as a single-scan imaging approach. These results align with findings from limited prior studies and underscore the need for validation in larger cohorts.

**Supplementary Information:**

The online version contains supplementary material available at 10.1007/s00259-025-07749-3.

## Introduction

Chronic bone and soft tissue infections (BSTIs) are a heterogeneous group of conditions, including periprosthetic joint infection (PJI), diabetic foot infection (DFI), chronic osteomyelitis (OM), and orthopaedic implant-associated infections. Their clinical relevance has increased substantially in recent years. The incidence of PJI parallels the increasing number of joint replacement surgeries in ageing populations, and the prevalence of DFI, a major complication of diabetes mellitus (DM), is increasing worldwide [1, 2]. Notably, these chronic infections often involve microorganisms with low virulence but high biofilm-forming capacity, enabling them to evade immune surveillance and persist despite treatment efforts [3].

Diagnosing chronic infections remains a clinical challenge. Due to the mild systemic inflammatory response, conventional clinical and laboratory parameters may lack sufficient sensitivity and specificity [4]. Imaging also presents limitations: MRI, although widely used in DFI and PJI, can be compromised by bone marrow oedema in Charcot neuroarthropathy (CNA) and by metal artefacts in prosthetic joints, causing false-positives [5, 6]. Nuclear medicine techniques such as [^99m^Tc]Tc-MDP three-phase bone scintigraphy (BS) offer high sensitivity but lack specificity [7]. [^99m^Tc]Tc-HMPAO-labelled leucocyte scintigraphy (LLS) may yield false negatives in chronic infections, as it predominantly targets neutrophils, whereas adaptive immune cells, particularly T lymphocytes, play a more prominent role in chronic infection, limiting the diagnostic accuracy of conventional LLS [8, 9].

Given these limitations of conventional scintigraphy, we hypothesized that CXCR4-targeted PET imaging (CXCR4 PET) using [^68^Ga]Ga-Pentixafor, which has shown promise in imaging various malignancies [10, 11], could provide better diagnostic accuracy owing to its specific binding to lymphocytes, particularly in chronic BSTIs, which are characterized by a lymphocyte-mediated adaptive immune response. However, CXCR4 expression is not restricted to infection and may also be increased in non-infectious inflammatory or reparative processes, which can limit specificity in certain settings.

## Materials and methods

### Study population and patient selection

This retrospective single-centre study included adult patients (> 18 years) with suspected bone and soft tissue infections (BSTIs), irrespective of aetiology, who underwent CXCR4 PET/CT as part of their diagnostic work-up. Accordingly, infected sites were categorized as periprosthetic joint infection (PJI), diabetic foot infection (DFI), chronic osteomyelitis, or orthopaedic implant-associated infection. All patients followed a fixed-sequence diagnostic workflow for suspected infection, beginning with [^99m^Tc]Tc-MDP three phase bone scintigraphy (BS), then [^99m^Tc]Tc-HMPAO-labelled leucocyte scintigraphy (LLS), and subsequently being scheduled for microbiological sampling or surgery. Consecutive patients who underwent CXCR4 PET (median: 7 days; range 2–12) within 2 weeks of completion of this workflow were enrolled in this study.

During this interval, patients’ clinical status was stable. Exclusion criteria included initiation of new systemic antibiotics or change in ongoing regimens, as well as acute infections, incomplete imaging workflow, or sites lacking an anticipated reference standard, as summarized in Supplementary Fig. 1. Within each patient, orthopaedic implants and diabetic foot regions without clinical suspicion of infection were included to provide internal controls, enabling comparison of tracer behaviour in non-infected tissue. Additionally, one acute fracture and one CNA region, both clinically suspected of superimposed infection, were also evaluated, reflecting real-world referral patterns and enabling assessment of [^68^Ga]Ga-Pentixafor uptake in conditions known to generate false-positive findings on scintigraphy.

The reference standard relied exclusively on microbiological culture and/or histopathological confirmation obtained during surgery or sampling, ensuring definitive classification of infected sites. For periprosthetic joint infections, these tissue-based criteria are congruent with the high-specificity “infection confirmed” category of the 2021 EBJIS definition of PJI. In contrast, non-infected control sites were characterised using clinical assessment, laboratory parameters (CRP, ESR, white blood cell count [WBC]), imaging findings, and ≥ 12 months clinico-radiological follow-up. Additional imaging (MRI or bone marrow scan) was documented when available.

The study was conducted in accordance with the principles of the Declaration of Helsinki, with ethical approval obtained from the Ethics Committee of Istanbul University Faculty of Medicine (Date: 13.03.2024/No: 2477682). Written informed consent was obtained from all participants prior to inclusion.

### [^68^Ga]Ga-Pentixafor PET/CT imaging

[^68^Ga]Ga-Pentixafor was synthesised according to a previously published protocol [12], using 25 µg of non-radioactive Pentixafor precursor (INN: boclatixafortide). After 60 ± 10 min following intravenous injection of 1.85–3.7 MBq/kg [^68^Ga]Ga-Pentixafor, whole-body PET/CT (Discovery IQ, GE Healthcare) was performed, including low-dose CT (140 kV, 50 mAs, 4 min/bed).

### [^99m^Tc]Tc-HMPAO labelling and scintigraphic imaging

Leucocyte labelling was performed in accordance with EANM guidelines [13]. After 4 h following intravenous injection of 555 ± 74 MBq [^99m^Tc]Tc-HMPAO, whole-body images were acquired (12 cm/min). For three-phase BS, 740 MBq [^99m^Tc]Tc-MDP were administered intravenously; blood-flow (2 s/frame, 60 frames) and blood-pool (1 min/frame, 5 frames) acquisitions were followed by whole-body and planar scans at 3 h. SPECT/CT acquisitions (20 s/frame, 60 frames, 128 × 128 matrix) were performed for both protocols using a hybrid system (Discovery NM/CT 670, GE Healthcare) with low-energy high-resolution collimators and low-dose CT (70 mA, 120 kV, pitch 1.375, rotation 0.5 s, slice thickness 1.25 mm).

### Imaging interpretation

Two nuclear medicine specialists, blinded to clinical data, jointly assessed scintigraphy (BS + LLS) and PET images side by side, resolving any differences during the same session. Image interpretation and quantitative measurements were therefore performed in consensus; thus, inter-observer variability was not assessed. Infection was defined as increased [^99m^Tc]Tc-MDP uptake on delayed-phase BS with corresponding focal leucocyte accumulation on LLS. Examinations were negative if no abnormal uptake was seen on either modality or if [^99m^Tc]Tc-MDP uptake lacked leucocyte accumulation. For foci with leucocyte accumulation but no [^99m^Tc]Tc-MDP uptake, bone marrow scintigraphy (BMS) was performed: concordant uptake indicated physiological marrow activity, while discordant uptake suggested infection.

Scintigraphic images were also graded using a simple 3-point visual uptake scale (1 = mild, 2 = moderate, 3 = intense, corresponding to ‘+’, ‘++’, and ‘+++’ in Table 2). For the head-to-head comparison, [^68^Ga]Ga-Pentixafor PET/CT images were evaluated visually and semiquantitatively. Tracer uptake at the site of suspected infection clearly exceeding the surrounding background was considered positive. Quantitative measurements were obtained in consensus by the two readers, comprising SUVmax (normalized to body weight) measurements in infectious foci and contralateral normal tissue, with target-to-background ratios (TBR) calculated.

#### Statistical analysis

The primary clinical endpoint was the diagnostic performance (sensitivity, specificity, positive (PPV) and negative (NPV) predictive values, accuracy, and receiver operating characteristic (ROC) area under the curve (AUC)) of CXCR4 PET compared with scintigraphy for detecting chronic BSTIs. Sensitivity, specificity, accuracy, PPV and NPV were calculated and are reported with 95% confidence intervals derived using the exact binomial (Clopper–Pearson) method. Secondary endpoints included diagnostic performance in the PJI and DFI subgroups and correlations between PET quantitative parameters (SUVmax, TBR) and inflammatory markers (CRP, ESR, WBC). Statistical analyses were performed using MedCalc (MedCalc Software Ltd, Belgium) and IBM SPSS version 30.0.0. Correlations between PET/CT quantitative parameters and laboratory results were assessed using Spearman or Pearson correlation analyses, as appropriate. A p-value < 0.05 was considered statistically significant. ROC analysis was conducted to compare diagnostic performance, with AUC values calculated for each modality. Differences in diagnostic performance between modalities were assessed by comparing correlated ROC curves using DeLong’s test. 

## Results

### Study cohort and reference standard

We evaluated 20 patients (10 men, 10 women; mean age 58 years [25–79]) with 25 suspected infectious foci and 14 control sites; baseline laboratory values and the distribution of suspected sites are summarised in Table 1.

**Table 1:** Patient characteristics

Total patients (*n*)	20	
Mean Age (Range)	58 (25–79)	
Gender		
Female (n)	10	
Male (n)	10	
Suspected Site of Infection		
Prosthesis (n)	9	
Diabetic Foot (n)	5	
Other (n)	6	
Laboratory Data		
Mean CRP (Range)	15 mg/L (1–67)	
Mean ESR (Range)	24 mm/h (4–57)	
Mean WBC (Range)	7.3 × 10³/µL (3.5–9.7 × 10³/µL)	

Infection was confirmed in 21/25 suspected foci: 8 foci (from 7 patients) were confirmed by concordant microbiological culture and postoperative histopathology, and 13 foci (from 10 patients) by microbiological culture alone. The remaining four suspected foci were classified as non-infected based on clinical and radiological follow-up. All control sites remained infection-free on clinical follow-up, laboratory evaluation, and imaging.

## Overall diagnostic performance

On visual interpretation, conventional scintigraphy (bone scintigraphy plus labelled leucocyte scintigraphy) correctly identified 19/21 infected sites (sensitivity 90%). Two sites were false-negative (one hip periprosthetic joint infection and one diabetic foot infection). Three sites were false-positive (suspected fracture, Charcot neuroarthropathy, and one intramedullary nail with increased uptake attributed to bone marrow expansion on bone marrow scintigraphy), yielding 15 true-negative sites overall.

[68Ga]Ga-Pentixafor PET/CT (CXCR4 PET) detected all 21 infected sites (sensitivity 100%). Two false-positive sites were observed (Charcot neuroarthropathy and the suspected fracture), whereas the intramedullary nail was correctly classified as a true-negative; overall, 16 sites were true-negative on CXCR4 PET (Table 2). Mean SUVmax in infected sites was 4.2 (range 1.7–7.8), and mean target-to-background ratio (TBR) was 5.7 (range 2.7–9.8).

**Table 2:** Clinical and imaging findings of patients evaluated for suspected infection

Category	Patient No.	Foci No.	Location	Scintigraphy Uptake	PET Uptake	SUVmax	TBR	Diagnostic Method	Pathogen	Antibiotic
Periprosthetic Joint Infections										
	1	1	THP	+/++	+++	4.6	5.8	Culture	Corynebacterium	+
	2	2	TKP	+++	+++	7.8	9.8	OTC	MRCoNS	+
	3	7	THP	+	+++	4.2	5.2	Culture	α-Streptococcus	–
	3	8	THP	FN	++	3.8	4.8	Culture	α-Streptococcus	–
	4	9	THP	++/+++	+++	6.6	8.2	Culture	MRCoNS	–
	5	11	THP	TN	TN	–	–	OTC	–	–
	6	13	TKP	+++	+++	4.5	5.6	Culture	K. pneumoniae	+
	7	15	THP	+/++	++/+++	4.9	7	Culture	MRSA	+
	8	17	THP	+	+++	6.3	7.9	OTC	P. aeruginosa	–
	9	18	THP	+	++	5.8	8.3	Culture	MRCoNS	–
Diabetic foot infections										
	10	19	DF	+++	++	3.6	5.1	Culture + MRI	Corynebacterium + P. aeruginosa	–
	10	20	DF	+	++	3.5	5.8	Culture	Corynebacterium + P. aeruginosa	–
	11	21	DF	+	++	4	5.7	Culture + MRI	K. pneumoniae + Corynebacterium	+
	11	22	DF	FN	+	3.5	5	Culture	K. pneumoniae + Corynebacterium	–
	12	23	DF	+	++	3.4	5.7	Culture + MRI	P. aeruginosa	+
	13	25	DF	+++	++	4	6.7	OTC + MRI	MRCoNS	–
	14	28	DF	+++	++/+++	3.4	4.3	OTC + MRI	β-Streptococcus	+
Osteomyelitis										
	15	30	OM	TN	TN	–	–	Follow-up	–	–
	16	31	OM	++	++	2.9	4.1	Culture	MSSA	–
	17	32	Maxillary OM	+++	+	2.7	2.7	OTC	K. pneumoniae + P. aeruginosa	+
Other Infections										
	2	6	Orthopaedic Plate	++	+	4.3	5.4	OTC	MRCoNS	+
	18	33	STI	+++	++	3	3.75	OTC	MRSA	+
Controls										
	2	3	TKP	TN	TN	–	–	Follow-up	–	–
	2	4	THP	TN	TN	–	–	Follow-up	–	–
	2	5	Vertebral instrumentation	TN	TN	–	–	Follow-up	–	–
	4	10	THP	TN	TN	–	–	Follow-up	–	–
	5	12	THP	TN	TN	–	–	Follow-up	–	–
	6	14	TKP	TN	TN	–	–	Follow-up	–	–
	7	16	THP	TN	TN	–	–	Follow-up	–	–
	12	24	DF	TN	TN	–	–	Follow-up	–	–
	13	26	DF	TN	TN	–	–	Follow-up	–	–
	14	29	DF	TN	TN	–	–	Follow-up	–	–
	18	34	IMN	TN	TN	–	–	Follow-up	–	–
	19	35	IMN	TN	TN	–	–	Follow-up	–	–
	19	36–37	IMNx2	TN	TN	–	–	Follow-up	–	–
Special Cases										
	13	27	CNA	++ (FP)	+ (FP)	1.7	2.8	MRI + BMS	–	–
	20	38	IMN	FP	TN	–	–	BMS + Follow-up	–	–
	20	39	Acute fracture	FP	FP	2.8	2.5	Follow-up	–	–

PET Visual Uptake score was descriptive only and was not used as a diagnostic cut-off

+ = faint uptake just above background or the contralateral side (score 1)

++ = clearly increased uptake (score 2)

+++ = markedly intense uptake (score 3). The visual score

*THP* Total Hip Prosthesis, *TKP* Total Knee Prosthesis, *DF* Diabetic Foot, *CNA* Charcot Neuroarthropathy, *STI* Soft Tissue Infection, *IMN* Intramedullary Nail, *TN* True Negative, *FN* False Negative, *FP* False Positive, *OTC* Operative Tissue Culture

Detailed case-anchored explanations for false-positive findings in acute fracture and Charcot neuroarthropathy are provided in Supplementary note 1.

**Fig. 1:**
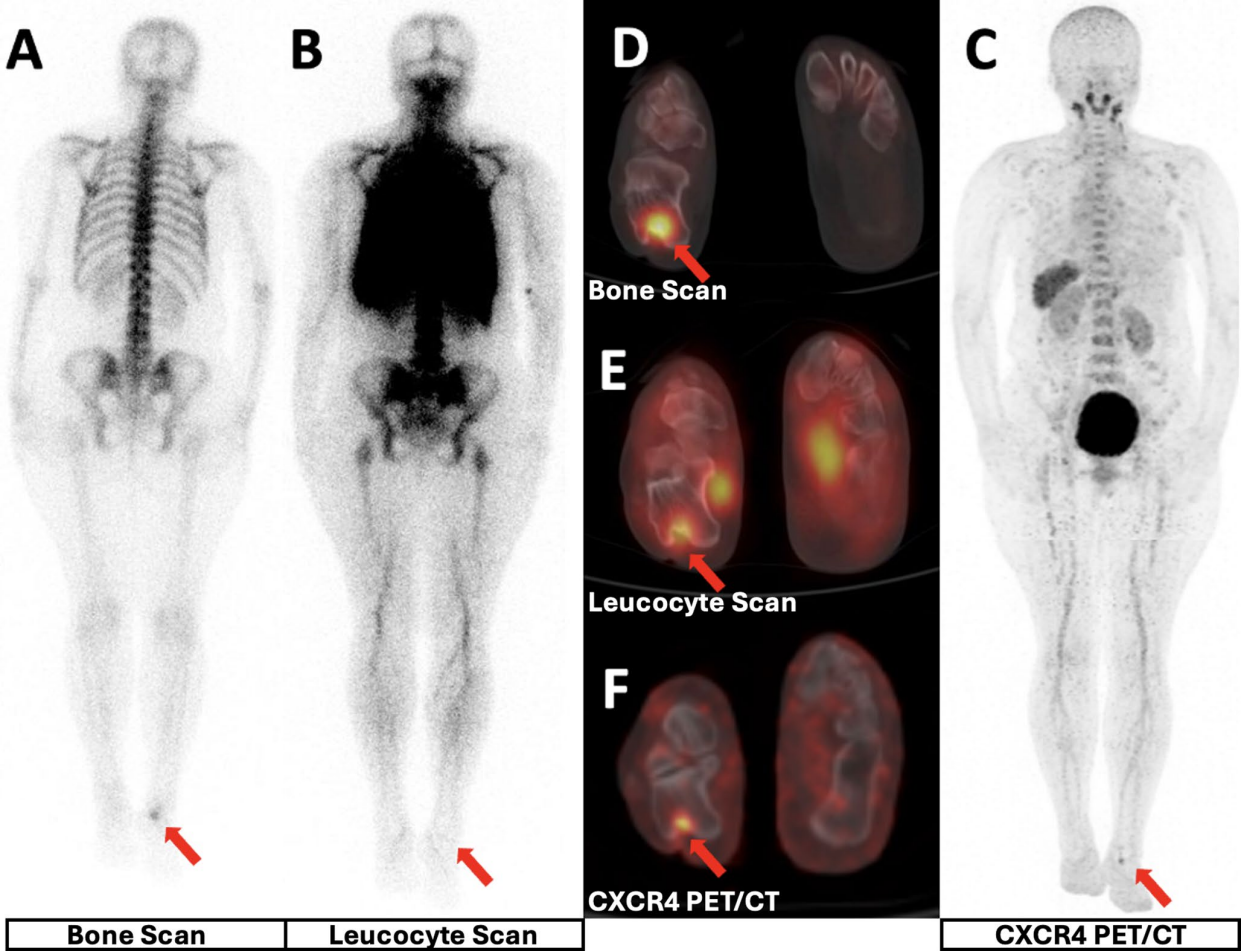
44-year-old female with chronic right heel exudate. BS (A, D), LLS (B, E), CXCR4 PET (C, F) show focal uptake at the posterior calcaneus with cortical destruction. Culture: MSSA. All modalities are true positives.

For the detection of chronic bone and soft tissue infections, diagnostic performance metrics for both modalities are provided in Table 3. In brief, CXCR4 PET demonstrated higher sensitivity and specificity than scintigraphy (100% vs. 90% and 89% vs. 83%, respectively), with higher overall accuracy (95% vs. 87%) and higher AUC on ROC analysis (0.94 (95% CI: 0.82–0.99) vs. 0.87 (95% Cl: 0.72–0.96)); however, the AUC difference did not reach statistical significance (*p* = 0.07) (Fig. 1).

**Table 3:** Diagnostic performance of scintigraphy and [^68^Ga]Ga-Pentixafor PET/CT

	Sensitivity (95% CI)	Specificity (95% CI)	Accuracy (95% CI)	PPV (95% CI)	NPV (95% CI)	AUC (95% CI)
Scintigraphy	90% (69–98)	83% (59–96)	87% (73–96)	86% (65–97)	88% (64–98)	0.86 (0.72–0.96)
PET	100% (84–100)	89% (65–99)	95% (83–99)	91% (72–99)	100% (79–100)	0.94 (0.82–0.99)

### Laboratory markers and PET quantitative parameters

Among patients with confirmed infection, elevated CRP and ESR were present in 7/16 (44%) and 11/16 (69%) patients, respectively, whereas leucocytosis was observed in 1/16 (6%). CRP correlated moderately with SUVmax (*r* = 0.54, *p* = 0.015) and TBR (*r* = 0.455, *p* = 0.038), while no significant correlations were observed between SUVmax/TBR and ESR or WBC.

### Subgroup analyses

#### Periprosthetic joint infection (PJI)

**Fig. 2:**
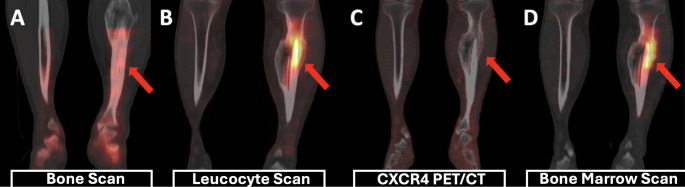
25-year-old male with prior tibial fracture and recent tarsal fracture for suspected osteomyelitis. BS normal (A); LLS uptake around intramedullary nail localized to marrow (B); CXCR4 PET no uptake (C); BMS confirmed marrow expansion (D). Labs normal. LLS = false positive; CXCR4 PET = true negative. One-year follow-up stable, consistent with reactive marrow expansion.

**Fig. 3:**
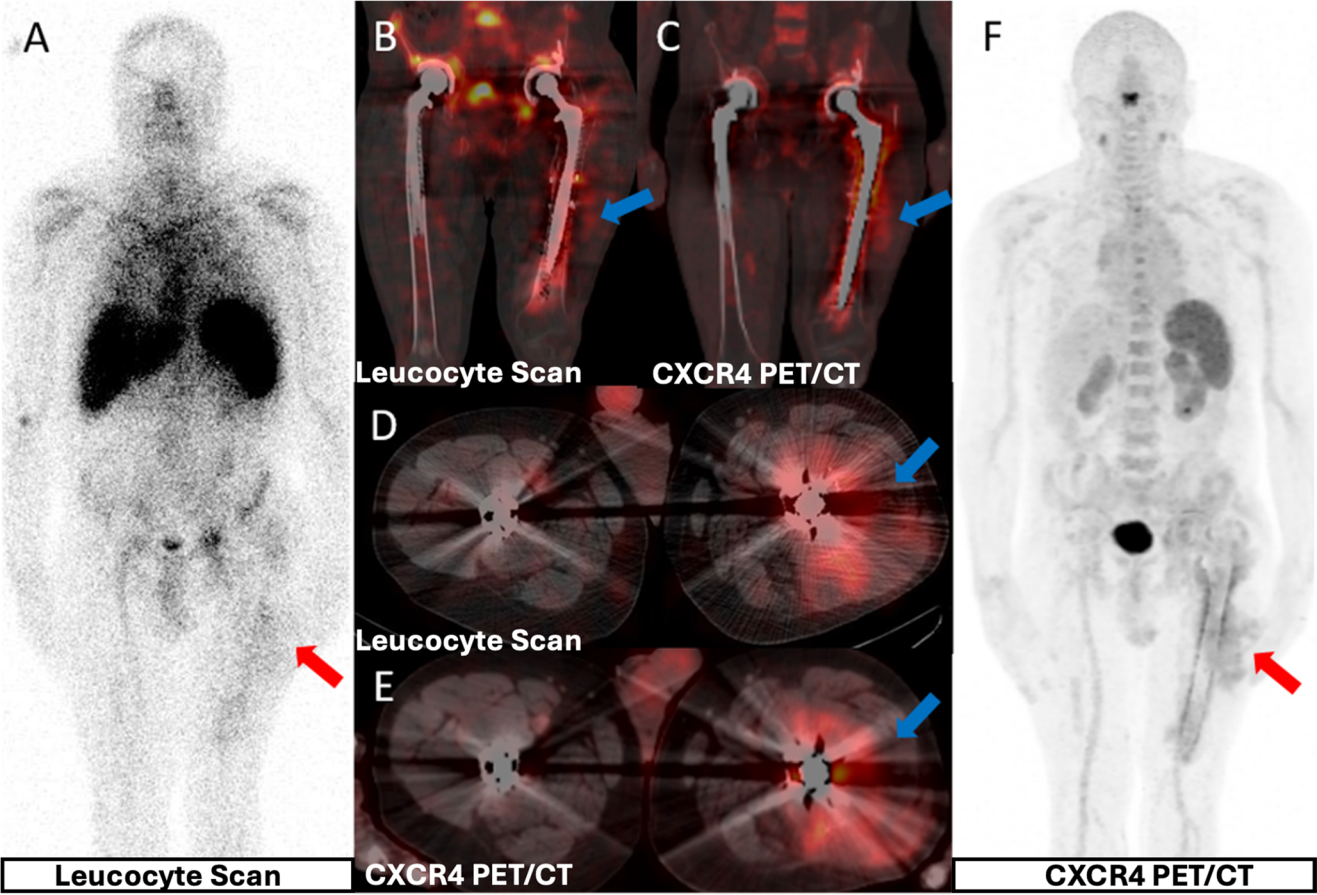
48-year-old male with ankylosing spondylitis and FMF with left THP pain. LLS (**A**, **B**, **D**) shows leucocyte accumulation around the prosthesis and CXCR4 PET (**C**, **E**, **F**) shows higher uptake. Culture: MRCoNS. Right THP asymptomatic and negative on both modalities (true-negative)

CXCR4 PET demonstrated pathological uptake in all infected prosthetic joints. Scintigraphy was false-negative in one infected hip PJI and positive in the remaining infected prostheses. All control prostheses (4 total hip prostheses and 2 total knee prostheses) were true-negative on both modalities. Consequently, CXCR4 PET achieved 100% sensitivity, specificity, accuracy, PPV, and NPV for PJI, whereas scintigraphy achieved 89%, 100%, 94%, 100%, and 88%, respectively (Fig. 2 and Fig. 3).

#### Diabetic foot infection (DFI)

**Fig. 4:**
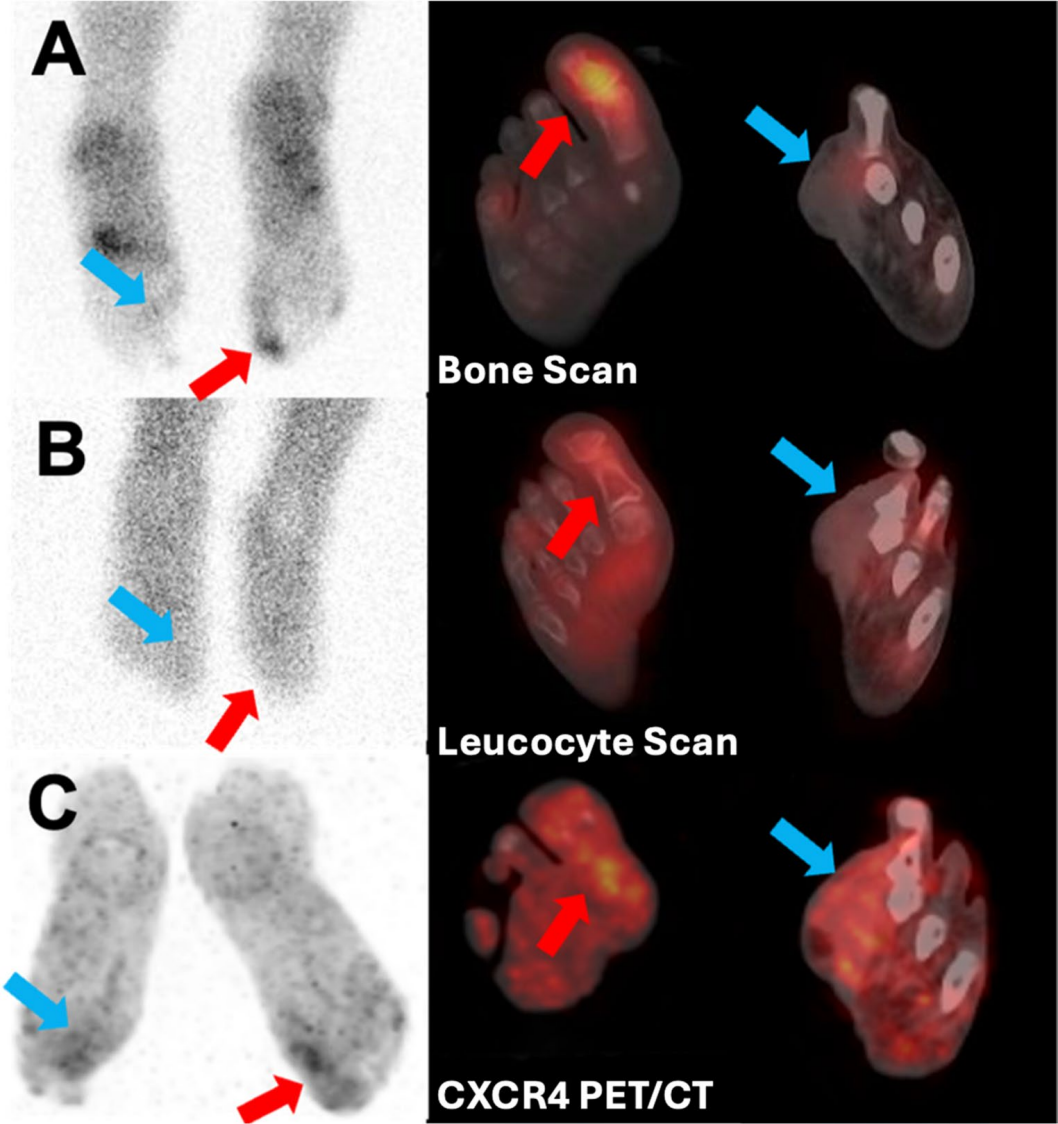
58-year-old male with diabetes and prior toe amputations. BS shows (**A**) focal uptake in left first toe (red arrows); LLS (**B**) mild leucocyte uptake; CXCR4 PET (**C**) significant uptake. Right foot: CXCR4 PET uptake suggesting soft-tissue infection despite absent WBC accumulation (blue arrows). Cultures: P. aeruginosa and Corynebacterium. Degenerative metatarsal changes caused heterogeneous BS activity

**Fig. 5:**
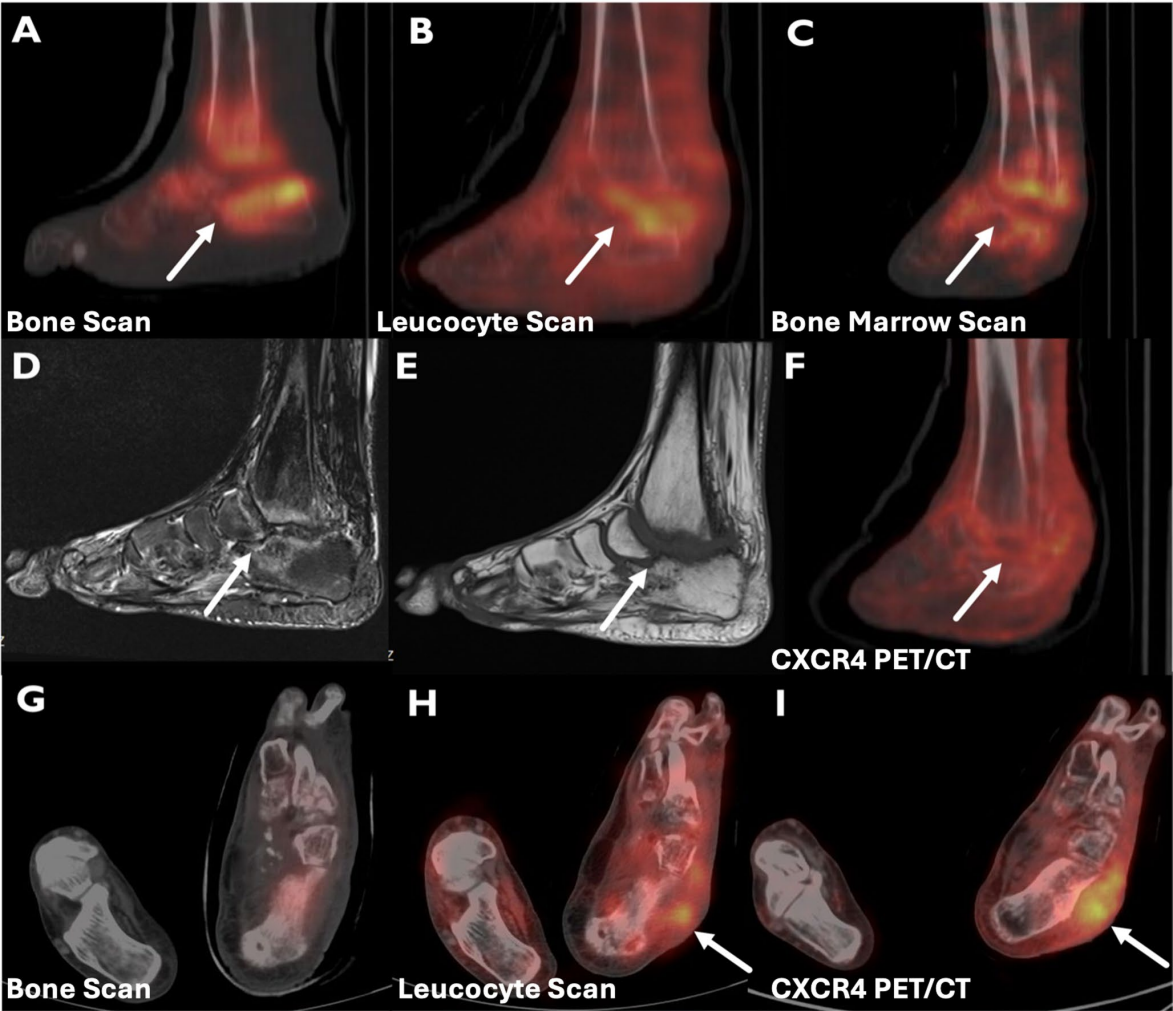
35-year-old male with diabetes and left foot chronic (6 years) neuropathic arthropathy. BS (A), LLS (B), BMS (C) increased uptake in tibiotalar and tarsometatarsal joints consistent with CNA; MRI T2 hyperintensity/T1 hypointensity (D, E). CXCR4 PET mild heterogeneous uptake in the CNA region (F). Lateral talar soft tissue uptake on LLS (H) and CXCR4 PET (I) but not on BS (G), suggesting soft tissue infection; culture: MRCoNS. No OM on follow-up; patient underwent arthrodesis for CNA.

In the DFI cohort, all 8 foci across 7 diabetic feet were ultimately classified as infected (5 combined osteomyelitis and soft-tissue infection; 3 isolated soft-tissue infection) (Fig. 4). CXCR4 PET showed increased uptake in all infected DFI foci; scintigraphy was positive in 7 foci and false-negative in 1 soft-tissue infection focus. One Charcot neuroarthropathy site demonstrated false-positive uptake on both modalities (Fig. 5). All control diabetic feet (*n* = 3) were true-negative on both scintigraphy and CXCR4 PET. Diagnostic performance for DFI is reported in Table 3 (scintigraphy: 88%/75%/83%/88%/75%; CXCR4 PET: 100%/75%/92%/89%/100% for sensitivity/specificity/accuracy/PPV/NPV, respectively).

#### Visual uptake patterns across entities

**Fig. 6:**
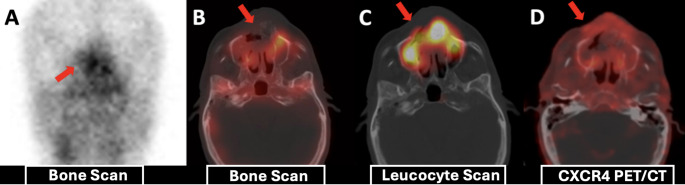
69-year-old male with a dental implant and peri-implant discharge. CRP 46 mg/L; ESR 57 mm/h; WBC 8.7×10³/µL. BS no osteoblastic activity (A, B); LLS intense leucocyte accumulation (C); CXCR4 PET milder uptake (D). Surgical culture: Klebsiella pneumoniae with necrotizing inflammation and neutrophilic abscess.

In subgroup-level visual comparisons, all seven hip PJI foci demonstrated higher uptake on CXCR4 PET than on scintigraphy, whereas the two knee PJI foci and one chronic osteomyelitis focus showed comparable uptake across modalities. Among six DFI foci that were true-positive on both techniques, scintigraphy demonstrated more intense uptake in four. Scintigraphy also showed higher tracer accumulation than CXCR4 PET in one case each of soft-tissue infection, maxillary osteomyelitis, and orthopaedic plate infection (Fig. 6).

## Discussion

Chronic BSTIs are increasingly prevalent and often present an indolent clinical course compared with acute infections. Chronic OM, frequently caused by low-virulence, biofilm-forming bacteria, is challenging to diagnose due to the frequent false-negative microbiological culture results in routine practice [14]. Orthopaedic implants carry a lifetime infection risk of ≈ 5%, which is heightened by revision surgery, obesity, prolonged procedures, advanced age, and DM [15]. Diabetic patients are prone to infections, especially DFI, which may involve both bone and soft tissue. DFIs account for ≈ 20% of hospitalizations and can progress to sepsis, requiring prolonged treatment [2]. Clinical assessment, laboratory tests, microbiological-histological analyses, and imaging techniques each provide valuable information. However, none alone achieves sufficient diagnostic accuracy [16, 17]. Among imaging modalities, LLS remains the most widely employed nuclear medicine technique for evaluating BSTIs. However, its practical application is constrained by venous blood sampling, complex laboratory labelling procedures, and prolonged acquisition times [18]. Furthermore, physiological uptake in the bone marrow can generate false-positive findings, necessitating additional BS or BMS, which incurs additional time, cost, and patient radiation exposure [19]. In addition, false negativity in chronic low-grade infections underscores the need for improved imaging strategies [16, 20, 21].

Our study evaluated the diagnostic performance of CXCR4 PET in comparison with scintigraphy as a potential single-scan PET/CT approach capable of simplifying the current multi-step scintigraphic workflow for detecting chronic BSTIs. Sensitivity and specificity were higher for CXCR4 PET than for scintigraphy (100% vs. 90% and 89% vs. 83%, respectively), which resulted in a higher AUC (0.94 vs. 0.86) on ROC analysis. These findings justify further investigation, although statistical significance was not achieved, likely due to the limited cohort size (*p* = 0.07). Notably, to our knowledge, this is the first study to assess CXCR4 PET in DFIs, thereby extending its evidence base beyond chronic OM.

Prior to our study, there was only one study by Bouter et al. in the literature, focusing solely on chronic OM, which also reported no false negatives [22]. Quantitative parameters were also in line with our findings (mean SUVmax and TBR 4.2 and 5.7 versus 3.3 and 8), and both were at visually favourable levels. Our study additionally pronounced the correlations between CRP and quantitative parameters, further suggesting a potential role for CXCR4 PET in therapy monitoring, although prospective follow-up data are needed. Furthermore, a 2018 study confirmed CXCR4 expression on OM tissue, originating predominantly from post-migratory T lymphocytes, underlining the specificity and reliability of CXCR4 PET [23].

### Periprosthetic joint infection

PJI, a subset of chronic BSTIs, has a distinct pathogenesis. Sinus tract, fever, and erythema are specific but insensitive, whereas pain and restricted mobility are sensitive but also common in aseptic loosening [24]. Mostly employed tests are CRP, WBC, ESR, and synovial fluid markers, which may yield false-positivity in non-infectious inflammatory conditions or false-negativity in low-grade infections, limiting their diagnostic reliability [16]. Microbiological culture and histology remain valuable, though prior antibiotic exposure, biofilm formation, and tissue heterogeneity may lead to false-negativity, while contamination can result in false-positivity [25]. Radiological imaging is not included in the latest EBJIS criteria, whereas nuclear medicine techniques are recommended. BS has high NPV, aids in excluding PJI, but positive LLS cannot independently confirm infection alone, placing patients in the “infection likely” group, a category for low-grade infections, of whom mostly do not fully meet the diagnostic cut-offs [26]. These limitations underscore the need for novel imaging modalities to refine diagnosis, particularly in indeterminate cases. Glithero et al. reported a sensitivity of 50% for LLS, highlighting the challenges of detecting chronic infection [21], whereas Kim et al. demonstrated that dual-phase LLS with SPECT/CT at 4 and 24 h achieved > 90% for all diagnostic parameters [27]. In our cohort, scintigraphy demonstrated sensitivity, specificity, accuracy, PPV, and NPV of 89%, 100%, 94%, 100%, and 88% for PJI, comparable to Kim et al., despite the absence of 24-hour imaging. Notably, CXCR4 PET correctly identified all prosthetic sites, whether infected or not, achieving 100% across all diagnostic parameters, highlighting its capacity to consolidate infection imaging into a single PET/CT examination. These findings are consistent with Bouter et al., who had no false-positive or negative results in their cohort of four patients with suspected PJI [22]. Moreover, CXCR4 PET identified a PJI missed by scintigraphy in a patient with normal CRP and ESR, and borderline WBC (4.3 × 10³/µL). Microbiological cultures of bilateral THP revealed alpha-haemolytic streptococci, capable of biofilm formation. The patient’s normal laboratory values reflect the often silent course of such infections [28]. This finding underscores the potential of CXCR4 PET to overcome false-negative findings in low-grade chronic PJI, beyond the sensitivity of LLS. 

In the literature, the most frequently reported cause of false-positive uptake on leucocyte scintigraphy (LLS) is physiological bone marrow activity [29]. This pitfall is exemplified in Fig. 2: intense LLS uptake was observed in marrow adjacent to an intramedullary nail after fracture fixation, with concordant activity on bone marrow scintigraphy (BMS), whereas CXCR4 PET demonstrated no uptake. The absence of clinical or laboratory signs of infection during one year of follow-up supports a false-positive LLS interpretation and a true-negative (TN) CXCR4 PET result. In this context, reactive marrow expansion related to osteogenesis and bone repair is a plausible explanation [30]. In contrast, two other sites in our cohort—an acute fracture and a CNA foot—showed increased uptake on both LLS and [^68^Ga]Ga-Pentixafor PET despite the absence of clinical or microbiological evidence of infection during long-term follow-up; diagnostically, these regions were therefore retained as false positives. This pattern is biologically plausible: sterile but intensely reparative environments (fracture callus and neuropathic joints) are characterized by hypervascularity, marrow expansion, and chronic synovitis, driving accumulation of both labelled leucocytes and CXCR4-expressing immune/progenitor populations [31–34]. While LLS predominantly reflects neutrophil trafficking, [^68^Ga]Ga-Pentixafor PET depicts a broader spectrum of CXCR4-positive cells (including lymphocytes, monocytes, and hematopoietic progenitors) involved in bone and joint remodelling [35–37]. Moreover, the CXCR4/SDF-1 axis is upregulated in fracture healing and neuropathic bone disease, mediating recruitment to sites of tissue injury [34, 38]. Collectively, these observations suggest CXCR4 PET may reduce marrow-related false positives, yet focal uptake in reparative regions must be interpreted with structural imaging and clinical trajectory—an essential consideration when differentiating low-grade infection from sterile hyperinflammatory remodelling in chronic BSTIs [13, 31, 32].

### Diabetic foot infection

In DFO management, the latest IWGDF/IDSA guidelines recommend MRI as the first-line imaging modality, with LLS and [^18^F]FDG PET/CT considered as alternatives [17]. However, their specificity decreases in bone marrow oedema, synovial effusion, or joint dislocation. A 2017 meta-analysis by Lauri et al., reported sensitivity and specificity of 93% and 75% for MRI, and 91% and 92% for LLS [39]. Furthermore, a 2020 multicentre study demonstrated that LLS specificity (92%) was significantly higher than that of MRI (71%) [40]. Sensitivity and accuracy were highest for LLS (76% and 86%). In STIs, LLS specificity exceeded 95%, while MRI specificity was 84%.

In our study, CXCR4 PET achieved 100% sensitivity, whereas LLS yielded a false-negative result in one STI patient, corresponding to a sensitivity of 88%, consistent with previous reports. Normal laboratory parameters in this patient suggest a low-grade inflammatory response. Specificity for both modalities was 75% in our study, which is lower than reported in the literature. The only false-positive finding in both imaging modalities was related to CNA. Notably, our study represents the first evaluation of CXCR4 PET in DFI patients.

### Additional observations

In the patient with maxillary OM shown in Fig. 6, both imaging techniques detected the infection, although LLS showed markedly higher uptake than CXCR4 PET. Histopathology revealed necrotizing inflammation and neutrophilic abscesses. Theoretically, necrosis-related hypoperfusion may have impaired lymphocyte migration and MDP accumulation, leading to reduced uptake on CXCR4 PET and BS, whereas preserved neutrophil diapedesis could still facilitate accumulation at the infection site [41]. A similar case was described by Bouter et al., classified as true-positive with a SUVmax of 3.7, slightly higher than our patient (SUVmax 2.7), though direct comparison is limited by the lack of histopathological and imaging details in that study [22].

The main limitations of our study include the small sample size, heterogeneous distribution of patients, the retrospective design, and the absence of post-treatment follow-up imaging. In addition, the clinical workflow in which CXCR4 PET/CT was performed only in patients proceeding to surgery or tissue sampling may have introduced selection and verification bias. Finally, image interpretation and SUV measurements were performed in consensus, and inter-observer variability was not assessed, which may limit the generalizability of the findings. Future prospective studies with larger cohorts are warranted to evaluate the utility of CXCR4-targeted PET imaging for monitoring therapeutic response.

## Conclusion

[^68^Ga]Ga-Pentixafor PET/CT demonstrated promising diagnostic accuracy in chronic BSTIs, detecting infection foci missed by conventional scintigraphy. It achieved 100% sensitivity and specificity in PJI in our cohort, with benefits including single-scan use, faster acquisition, and higher resolution. In chronic STIs, reduced uptake versus LLS may indicate impaired vascularity, while false positives in fractures and CNA highlight ongoing challenges with non-infectious inflammation. As the first study assessing its role in DFI and chronic STIs, [^68^Ga]Ga-Pentixafor PET/CT appears to be a promising diagnostic tool. In light of the study’s limitations, these hypothesis-generating preliminary findings warrant further validation in larger and more diverse patient populations.

## Supplementary Information

Below is the link to the electronic supplementary material.

Supplementary Material 1

Supplementary Material 2

## Data Availability

The datasets generated and/or analysed during the current study are available from the corresponding author on reasonable request.
