# Supplementary figures and images for: Evaluating chronic bone and soft tissue infections with [68Ga]Ga-Pentixafor PET/CT: a head-to-head comparison with scintigraphy
Source: Eur J Nucl Med Mol Imaging. 2026 Jan 27;53(5):3271–82. doi: 10.1007/s00259-025-07749-3 (PMC13013284; doi:10.1007/s00259-025-07749-3)

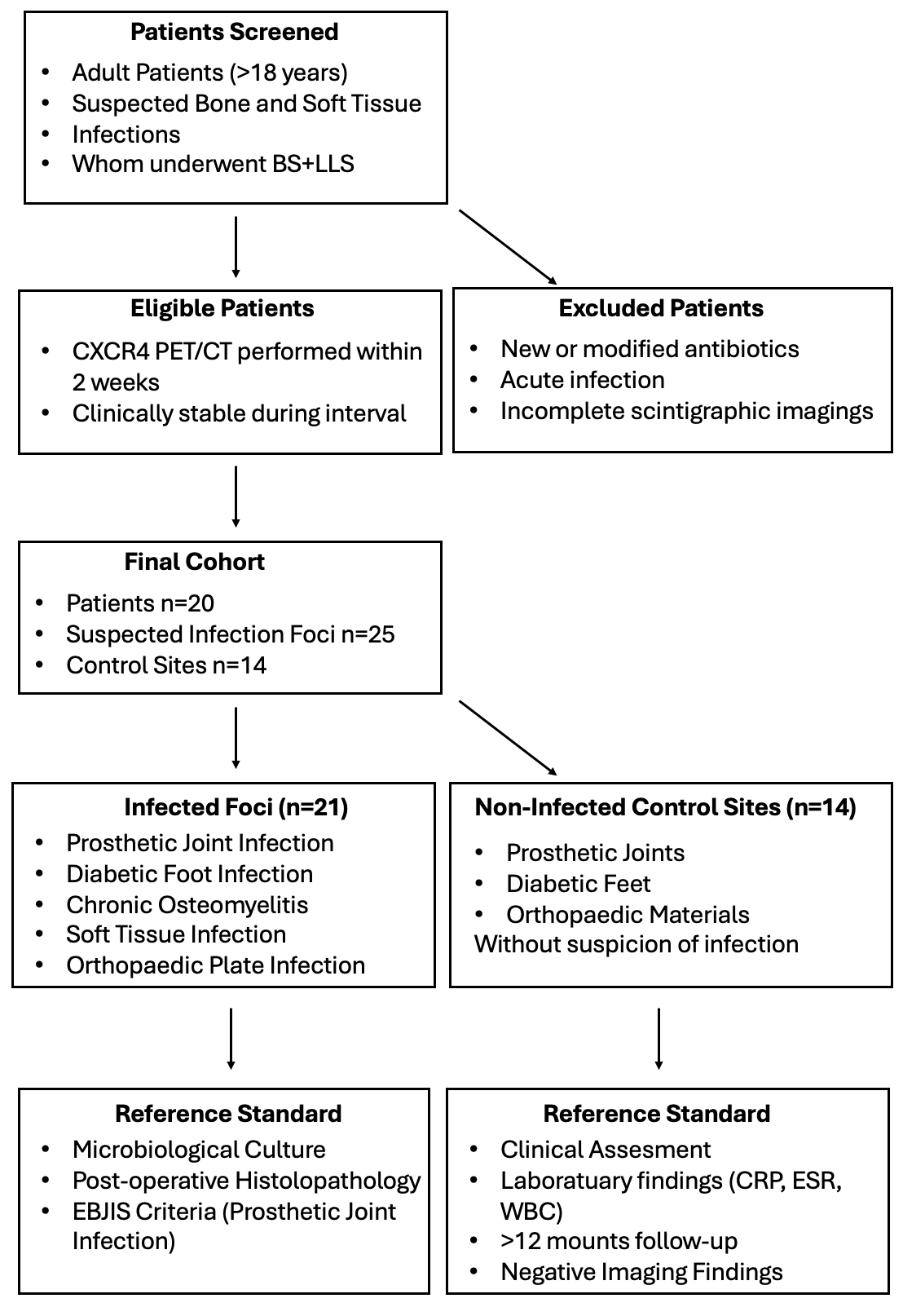

Supplement: Supplementary file 1 — Supplementary Material 1 [file 259_2025_7749_MOESM1_ESM.png]
