# Supplementary material for: Evaluating chronic bone and soft tissue infections with [68Ga]Ga-Pentixafor PET/CT: a head-to-head comparison with scintigraphy
Source: Eur J Nucl Med Mol Imaging. 2026 Jan 27;53(5):3271–82. doi: 10.1007/s00259-025-07749-3 (PMC13013284; doi:10.1007/s00259-025-07749-3)
Supplement: Supplementary file 2 — Supplementary Material 2 [file 259_2025_7749_MOESM2_ESM.docx]

**Supplementary Note 1. Case-anchored explanation of false-positive findings**

1. **Charcot neuroarthropathy case**

In the Charcot neuroarthropathy case, periarticular tracer uptake was observed consistently across bone scintigraphy, labeled leukocyte scintigraphy, and [^68^Ga]Ga-Pentixafor PET/CT, as illustrated in Figure 6. Subsequent bone marrow scintigraphy demonstrated concordant tracer uptake in the same periarticular region, suggesting reactive bone marrow involvement rather than infection.
 MRI revealed T2-hyperintense and T1-hypointense signal changes consistent with active Charcot neuroarthropathy, without imaging features suggestive of osteomyelitis.
 Based on the concordant multimodality imaging findings, the case was interpreted in favor of Charcot neuroarthropathy, and the patient was referred for arthrodesis. Intraoperative tissue cultures obtained during surgery showed no microbial growth, definitively excluding infection. Accordingly, the periarticular bone uptake observed on [^68^Ga]Ga-Pentixafor PET/CT, as well as on bone and labeled leukocyte scintigraphies, was classified as a false-positive finding.

1. **Acute fracture case**

In the acute fracture case, focal tracer uptake was observed on bone scintigraphy, labelled leucocyte scintigraphy, and [^68^Ga]Ga-Pentixafor PET/CT. **Although tissue sampling had initially been planned, the patient declined further invasive diagnostic procedures after PET/CT, and therefore, no sampling or** additional cross-sectional imaging was performed. The fracture site identified on CT was radiologically monitored over time.
 Over 12 months of clinical, laboratory, and imaging follow-up, no evidence of infection was identified. Stable fracture appearance with physiological callus formation on follow-up imaging supported classification of the observed tracer uptake on conventional scintigraphy and [^68^Ga]Ga-Pentixafor PET as a fracture-related inflammatory false-positive finding.
